# Active Tactile Sensibility of Brånemark Protocol Prostheses: A Case–Control Clinical Study

**DOI:** 10.3390/ma14164644

**Published:** 2021-08-18

**Authors:** Nathalia Moraes, Eduardo Moraes, Tiago Anastacio, Licínio Silva, Aldir Machado, José Schoichet, Raphael Monte Alto, Rafael Mello-Machado, Angelo Cardarelli, Carlos Fernando de Almeida Barros Mourão, Priscila Casado, Georgios Romanos

**Affiliations:** 1 Pos-graduation of Implant Dentistry, School of Dentistry, Universidade Federal Fluminense, Niteroi 24020-140, Brazil; nathalia_bm@hotmail.com (N.M.); tiagohenrique.mg@gmail.com (T.A.); aldirmachado@gmail.com (A.M.); jschoichet@terra.com.br (J.S.); raphaelmontealto@yahoo.com.br (R.M.A.); priscilacasado@id.uff.br (P.C.); 2 De Moraes Institution, Rio de Janeiro 22050-001, Brazil; moraes.edujm@gmail.com; 3 Implant Dentistry Department, Universidade Iguaçu, Nova Iguaçu 26260-045, Brazil; licinio.da.silva@gmail.com (L.S.); rafaelcoutinhodemello@yahoo.com.br (R.M.-M.); 4 Department of Dentistry, University Vita Salute San Raffaele, 20100 Milan, Italy; angelo_cardarelli@libero.it; 5 Clinical Research Laboratory in Dentistry, Universidade Federal Fluminense, Niteroi 24020-140, Brazil; 6 School of Dental Medicine, Stony Brook University, New York, NY 11794, USA

**Keywords:** prosthodontics, occlusion, clinical research, active tactile sensibility, dental implant

## Abstract

Few studies have assessed active tactile sensibility in patients rehabilitated with implants. Improved knowledge about functional tactile sensibility will contribute to several clinical applications, such as protocols for immediate loading, prosthesis design, occlusal improvement in implantology, and physiological integration of implant-supported prostheses. The present study evaluated active tactile sensibility in patients rehabilitated with Brånemark-type mandibular prostheses that impede the total mucosa-supported maxillary prosthesis. Thirty-five subjects participated in this study. The experimental group (*n* = 18) inclusion criteria were as follows: Brånemark-type prosthesis and a total mucosa-supported maxillary prosthesis. The control group (*n* = 17) was composed of participants with complete healthy dentition. Carbon foils with different thicknesses (12 μm, 24 μm, 40 μm, 80 μm, and 200 μm) were placed in the premolar region to evaluate the brink of active oral tactile sensibility. The researchers assessed the participants 120 times. After evaluation, we observed a statistical difference (*p* < 0.05) between the groups. Additionally, the degree of sensibility was found for all thicknesses, except for 12 μm, on both sides. There was a more significant increase in perception in the control group as the carbon thickness increased. The tactile sensibility threshold was 2.5 times greater for participants with prostheses. Thus, the tactile sensibility for mandibular implant-supported and maxillary mucosa-supported prostheses is significantly lower than that of dentate patients, which was detected above the thickness of 80 μm; in patients with natural dentition, different thicknesses were seen starting from 24 μm.

## 1. Introduction

Several studies have shown that implant-supported fixed prostheses are an excellent substitute for replacing lost teeth [1,2]. Despite the fact that patients who are rehabilitated with osseointegrated implants do not present significant impairment in their oral functions, the mechanism of compensation for lost periodontal ligament around the implants is not completely understood [3].

First of all, the tactile function of a tooth is diagnosed by the presence of periodontal ligament. Tooth extraction eliminates those sensitive periodontal mechanoreceptors directly impacting sensory feedback and, consequently, oral tactile function [3]. Even after prosthetic rehabilitation, whether with a conventional or implant-supported prosthesis, the tactile function remains jeopardized [4]. This fact may represent a subsequent risk of implant overloading [5].

The absence of periodontal ligament around the implants can lead to a biomechanical and neurophysiological deficit when compared to natural dentition [6]. Osseoperception is the name of the phenomenon that refers to the awareness of stimuli applied in patients with implant-supported prosthesis, and it has been described for both oral and skeletal implants [5]. Osseoperception was first identified by Torgny Haraldson, a pioneer researcher in dentistry, whose careful work with patients established the oral function of osseointegrated prostheses when investigating osseointegration. In particular, his study of bite force and oral function established the importance of sensory feedback control provided by osseointegrated prostheses [7].

However, even today, little is known about how much osseoperception can affect the psychophysical relationship of implants [8]. The osseointegration of implants in maxillary bones has been widely studied from the biomechanical, histological, and microbiological aspects. However, the physiological integration of implants and implant-supported prostheses has earned little attention [5].

The assessment of osseoperception can be studied noninvasively through psychophysical assessments. There is a distinction between the passive tactile sensibility threshold and active tactile sensibility threshold. Passive tactile sensibility consists of detecting the pressure threshold, that is, a passive stimulus applied not requiring the individual to perform any action [8]. Active tactile sensibility consists of the discriminatory ability to detect the thickness of objects placed between the teeth. In this test, the individual performs a physical action, such as occluding. Therefore, in partially and completely edentulous patients, with consequent loss of periodontal ligaments in the area, active and passive tactile sensitivities are altered even when they have been rehabilitated with implant-supported prostheses [9].

The proprioceptive response plays an essential function in the adjustment of fine motor control and modulation of complex mandibular movements, sensory discriminative capacities, and masticatory reflex [10]. In dentate individuals, sensory input can be provided by two different groups of mechanoreceptors: (1) remote fibers (which originate in the dental pulp temporomandibular joint, masticatory muscles, buccal mucosa, and periosteum) are responsible for the discrimination of large particles, and (2) proprioceptors in the periodontal ligament can detect a finer stimulus, depending on the specification of the direction, magnitude, and overload occlusal force [5,10,11].

Removal of proprioceptor fibers from the periodontal ligament after tooth extraction may impair this precise control. However, although they do not have a periodontal ligament, implants have shown much better tactile sensibility than soft tissue prostheses, implying a partial substitution of sensibility [12,13].

The phenomenon of osseoperception represents an essential step for functional and physiological integration of dental implants, and it is of significant interest to researchers in this field. Although such a mechanism is not fully understood, researchers suggest that osseoperception may result from mechanoreceptors in the remote nerve endings, periradicular tissues of the opposing teeth, cortical synaptic remodeling in the brain, or probable innervation around implants [4,10].

Such implant-mediated sensory–motor interactions can help achieve a more natural oral function with important clinical implications [10]. A highly sensitive implant can recover proper motor sensory control and, thus, increase chewing efficiency, improve inhibitory reflex response in masticatory muscles, prevent traumatic occlusion, and decrease the risk of overloading of remaining teeth and implants [5]. Therefore, assessing the efficacy of implants to discern tactile fine stimuli is of significant relevance [14].

However, to date, very few studies have assessed active tactile sensibility in patients rehabilitated with implants [5]. Improved knowledge about active tactile sensibility will contribute to several clinical applications, including occlusal adjustment in implantology, protocols for immediate loading, prosthesis design, implant survival, and physiological integration of implant-supported prostheses with the stomatognathic system. Therefore, the aim of the study was to assess the active tactile sensibility in patients rehabilitated with the Brånemark protocol prosthesis occluding on the total mucosa-supported prosthesis. The authors hypothesize that active tactile sensibility is lower in the supporting Brånemark protocol prosthesis.

## 2. Materials and Methods

This observational study was conducted in accordance with the Helsinki Declaration of 2013 for human research and Resolution number 466 of 12 December 2012 of the National Health Council and approved by the Research Ethics Committee involving humans of the School of Medicine of the Universidade Federal Fluminense, Niterói, RJ, Brazil, under report number 1.616.110. 

### 2.1. Selection of Participants

A total of 35 randomly selected participants from the Specialization Course in Implant Dentistry of the Dental School of the Fluminense Federal University agreed to participate in the study and signed a free and informed consent form.

**Figure 1:**
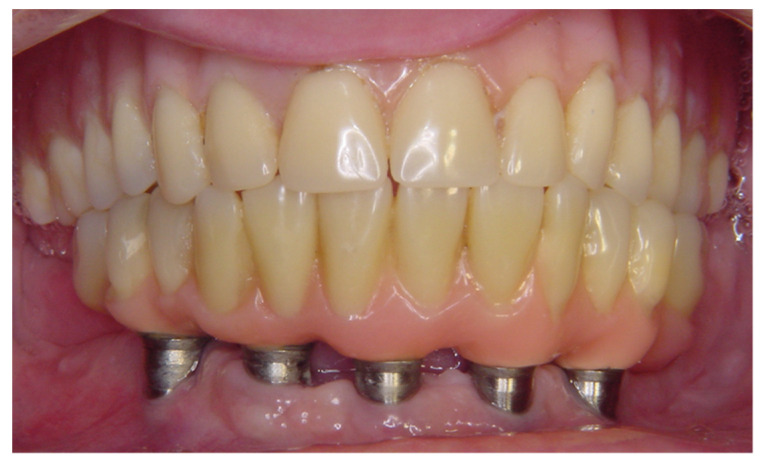
Clinical aspect of Brånemark protocol mandibular prostheses (experimental group), showing total implant-supported prosthesis.

**Figure 2:**
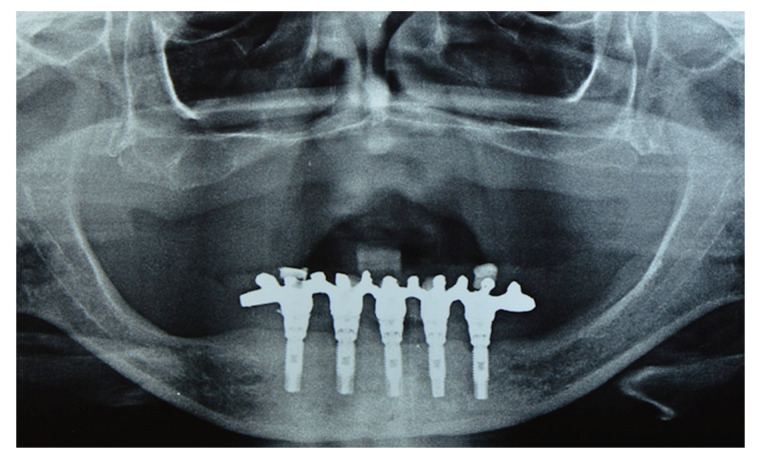
Radiographic aspect of Brånemark protocol mandibular prostheses (experimental group), showing external hexagon implants supporting a fixed mandibular prosthesis.

To be included in the experimental group (*n* = 18), the participants were to have a mandibular Brånemark protocol prosthesis (Figure 1 and Figure 2) and a total maxillary removable mucosa-supported prosthesis. The mandibular prostheses of all volunteers assessed in the study were made of acrylic resin, and the teeth were placed on a metal structure. The prostheses were made on conical mini pillars, which minimized situations such as a lack of passivity that could modify the results. Exclusion criteria were a history of temporomandibular disorder and/or bruxism, absence of canine guidance in occlusion, inadequate bone for implant anchorage revealed by a radiographic examination, signs of inflammation around implants, lack of occlusal stability, inadequate vertical dimension, fracture, or any signal of prosthesis wear. Participants who reported carbon or latex allergies, materials used in this experiment, were also excluded.

The control group (*n* = 17) was composed of volunteers who had complete dentition and who were not undergoing dental treatment. Exclusion criteria for the control group were as follows: participants with unsatisfactory dentoalveolar insertion, absence of canine guide in occlusion, those with signs and symptoms of temporomandibular disorder and bruxism, those who presented signs of gingival inflammation or periodontal disease, such as bleeding on probing, probing depth > 4 mm, tooth mobility, loss of clinical attachment level, those who had some type of restorative material in the premolar region, absence of canine guidance in occlusion, those undergoing orthodontic treatment, and participants who reported allergies to carbon or latex gloves used in the experiment.

There was no limitation regarding the age group. Any volunteer who met the inclusion and exclusion criteria could participate in the study. It is important to point out that participants who were on medication and had systemic diseases were not excluded from the research.

### 2.2. Active Tactile Sensibility

**Figure 3:**
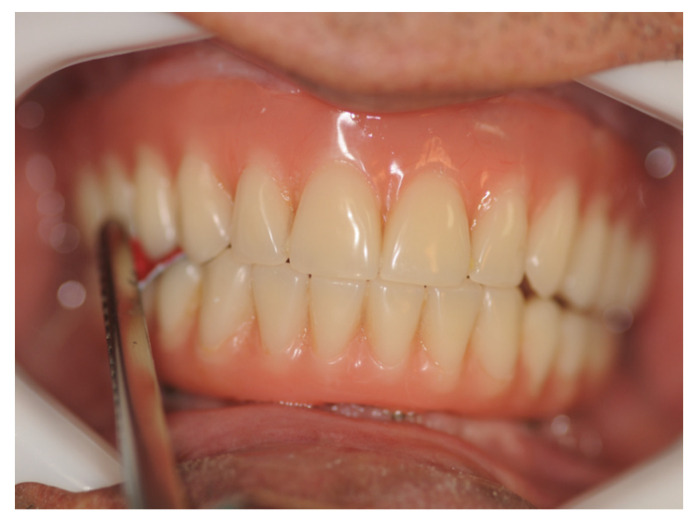
Evaluation of active tactile sensibility with carbon sheets in the premolar area of a participant with Branemark protocol prostheses (experimental group). The lip retractor facilitated access.

**Figure 4:**
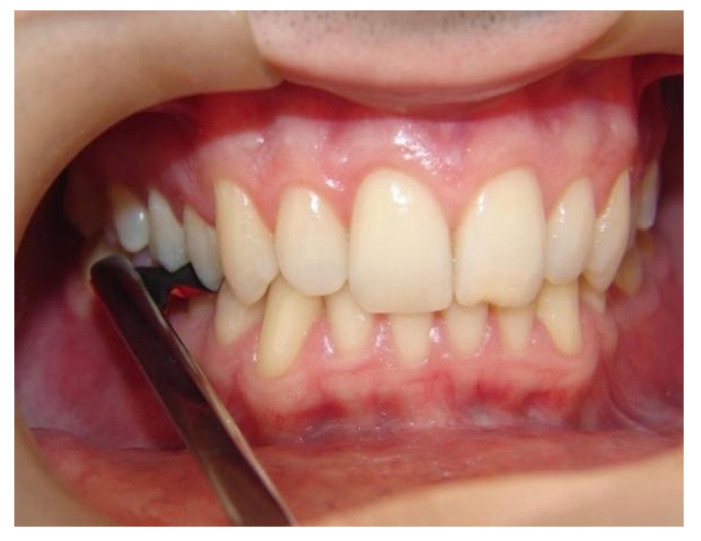
Evaluation of active tactile sensibility with carbon sheets in the premolar area of a participant (control group).

The oral active tactile sensibility threshold was evaluated with 10 cm × 7 cm sheets of carbon (BAUSCH, KG, Colonia, Germany) of different thicknesses (12 μm, 24 μm, 40 μm, 80 μm and 200 μm) and double color, which were placed in the premolar region of the participants assessed. The premolar region was chosen due to its high masticatory strength and straightforward access for the interposition of the sheet of carbon during the tests. A placebo test was performed by placing a Miller caliper without carbon, representing 0 μm. The researchers assessed the participants 120 times, 60 times on the right side and 60 times on the left side, totaling 10 tests on each side for each thickness and 10 placebo tests. The total duration of the assessment was about 40 min. The selection of thickness was performed randomly as the sheets of carbon were randomly selected for each occlusion, then becoming randomized as a function of not only the thickness of carbon but also the time of occlusion, until 10 tests for each side (right and left) were completed with each thicknesses and placebo. The study was double-blinded, that is, one examiner interposed the sheets of carbon between the participant’s arches while another examiner noted the results on the form for data collection. During the experiment, participants were blindfolded and sat upright in a quiet environment. The researchers used a lip retractor (MORELLI ORTODONTIA, Sorocaba, São Paulo, Brazil) to facilitate the interposition of the sheet of carbon in the region to be assessed, so as to prevent contact of the carbon with other areas such as cheeks, tongue, and lips. In addition, the carbon width was determined according to teeth diameter to be in contact only with the tooth surface, minimizing interferences that could modify the result. The sheets of carbon were inserted by examiner 1 with the help of Miller tweezers (GOLGRAN, São Caetano do Sul, São Paulo, Brazil), and, once the carbon was inserted in the premolar region, the participant was asked to occlude. Using gestures with the left hand, the participant informed examiner 2 whether the presence of the sheet of carbon was felt or not. Examiner 2 noted the results on a proper form for data collection. The tests were first performed on the right hemiarch and then on the left side, and each sheet of carbon was tested 10 times (Figure 3 and Figure 4). The placebo test using no sheet of carbon was also performed 10 times, totaling 60 tests for each hemiarch. Detection from the participant equal to or more than five times (50%) was considered positive, i.e., active sensibility perception.

### 2.3. Statistical Analysis

The threshold was based on the study of Enkling et al. [15], who established that the most accepted method for determining the active tactile sensibility threshold is a response level of 50% correct (positive) responses, i.e., a test with the highest reproducibility for this threshold. Therefore, all absolute values, in micrometers, for each response were considered when assessing the results of both groups.

According to the specific literature [3,15,16,17], the variances for the comparison between the groups were estimated, obtaining the estimation of about 17 participants per group at a confidence level of 5% and power of 80%, according to the sample calculation for the one-way analysis of variance. 

The Shapiro–Wilk test was applied to evaluate the normal distribution of data, and the Mann–Whitney test was applied to compare the percentage sensibility between groups. Values of *p* < 0.05 were considered significant. The Friedman test, at a 5% significance level, was applied to find the statistically significant differences (*p* < 0.05) in sensibility to the six thicknesses assessed, considering analysis within each group for each side.

## 3. Results

A total of 35 participants were included in the study, 17 in the control group and 18 in the experimental group, with a mean age of 46 ± 20.9 years, of which 24 were men and 11 were women. There was no statistically significant difference between the groups, considering age and sex (*p* > 0.05). Brånemark protocol prostheses were supported by a mean of 4 ± 2.1 implants. Experimental group data are shown in Table 1.

The results included the analysis of the right and left sides between the groups studied and the thickness variables (0 μm, 12 μm, 24 μm, 40 μm, 80 μm, and 200 μm), considering the percentage active tactile sensibility detected by each research participant in each group with a threshold of 50% positive responses.

### Active Tactile Sensibility between the Two Groups: Control and Experimental

**Figure 5:**
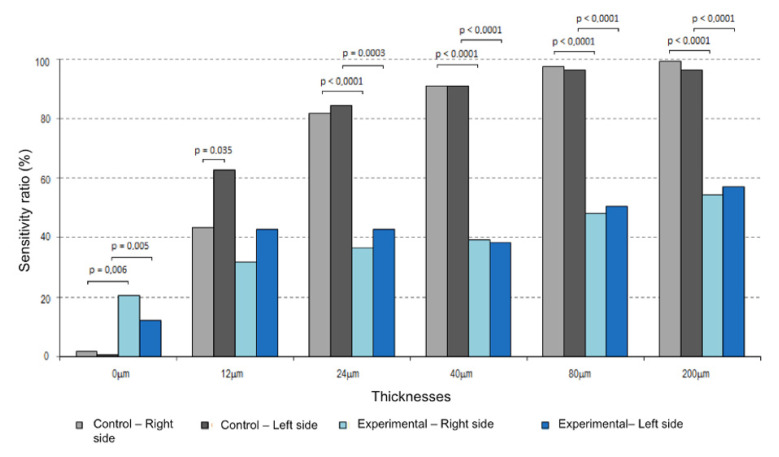
Differences between the groups studied, considering the thicknesses and *p*-values. Values of *p* < 0.05 were considered significant.

When comparing the two groups, it was found that, except for the 12 μm thickness, there was a statistically significant difference (*p* < 0.05) between the percentage sensibility in the control group and experimental group, on both the right and the left sides. In the experimental group, 27% of participants reported feeling the sheet of carbon during the placebo tests. Sensibility in the control group was above 50% (positive sensibility) for the thicknesses 24 μm, 40 μm, 80 μm, and 200 μm, in contrast to the experimental group, in which the values of active tactile sensibility were above 50% after a thickness of 80 μm. It was also found that there was a greater increase in perception on both sides in the control group as the carbon thickness increased in comparison to the experimental group, in which this perception did not increase. Table 2 summarizes the findings, and Figure 5 illustrates the differences.

The Friedman test, at a 5% significance level, was applied to show the statistically significant differences (*p* < 0.05) between groups in terms of sensibility to the six thicknesses, considering the analysis for each side. The difference was considered statistically significant (*p* < 0.003) after the application of Bonferroni correction for the significance of multiple thickness comparisons.

In the control group, there was active tactile sensibility on the right side with perception of most of the different thicknesses (*p* < 0.03). On the other hand, on the left side, the thickness sensibility was lower for thicknesses 12 μm and 24 μm (*p* > 0.03).

For the experimental group, perception of the carbon was detected on the right side, considering a threshold of 50% correct responses after a thickness of 80 μm. On the left side, this perception was evident for all thicknesses when compared to placebo (0 μm). However, in the experimental group, a difference between the right and left sides was not detected.

Considering the sensibility threshold, on the basis of the percentages of thickness detection on the right and left sides, it was found that the active tactile sensibility threshold was 2.5 times lower for prosthetic wearers than for dentate participants. This means that active tactile sensibility for participants with maxillary mucosa-supported and mandibular implant-supported prostheses is significantly lower in comparison with dentate participants, detected above a thickness of 80 μm, without any differentiation for thicknesses.

## 4. Discussion

Osseointegrated implants are widely used for the rehabilitation of edentulous patients. The osseointegration of implants in maxillary bones has been extensive studied from a biomechanical, histological, and microbiological point of view, but the physiological integration of implant-supported or -retained prostheses has received little attention [5,18]. Studies assessing active tactile sensibility in patients with implant-supported fixed complete dentures are rare [3,17,19]. Only one study of patients with implant-supported fixed complete acrylic resin restorations was found in the literature [3]. In the present study, it was found that patients rehabilitated with implant-supported fixed complete prosthesis showed a higher active tactile sensibility threshold than patients with natural dentition.

Mechanoreceptors in the buccal region are in the periodontal ligament, periosteum alveolar mucosa, tongue, bone, and gingiva. The mechanoreceptors located in the periodontal ligament contribute to the acute sensibility of teeth to mechanical stimuli [18]. The three most common types of sensitivities assessed are passive tactile sensibility, where a stimulus is applied passively on a tooth or implant, active tactile sensibility, where the patient perceives objects interposed between the antagonistic arches, and vibro-tactile sensibility (dynamic sensibility threshold), which is poorly researched [20].

Active tactile sensibility is a noninvasive procedure with clinical applicability that assesses the presence or absence of periodontal ligament receptors when teeth are present and detects muscle or bone receptors in patients with implants [5]. This is because purely passive stimuli are practically inexistent during mandibular movements of chewing, swallowing, and speaking [19].To assess active tactile sensibility, also called occlusal discrimination capacity, most studies use sheets of aluminum [3,21], copper [15,19,22,23], and even gold [14]. Only one study used plastic tapes during the assessment of active oral tactile sensibility in patients with indirect restorations [24].

The use of metallic materials during the assessment may mask the results, as metals present thermal conductivity [18]. Therefore, in our study, we chose to use sheets of carbon as it is a clinical material used for occlusal adjustments. In addition, we used different thicknesses produced by the same manufacturer to minimize biases related to the composition of the material.

During the tests, we decided that participants could not use headphones as we believed that it could be a distracting factor for the volunteers, corroborating with previous studies [3,14,17]. Furthermore, we used the same number of replicates and thicknesses in the placebo test, which differs from most other studies, as fewer assessments are reproduced when the placebo is applied [14,19]. Thus, we were able to establish the real absence of detection of interdental thickness in dentate patients, a difference that is almost imperceptible to patients with implant-supported prostheses.

The test was performed on the two hemiarches (right and left) of the patients to reduce the possibility of variations in the same individual. The thickness of the sheets of carbon was chosen randomly to avoid a patient learning curve, which could lead to false positive results when detecting thinner sheets.

Enkling et al. [15] stated that, according to the studies published to date, the tactile sensibility threshold can be determined in different ways: detection of the thinnest thickness identified, 50% limit of positive or correct responses [8,17,18,21], and 80% limit of positive or correct responses [24]. In our study, we chose to use the 50% limit of positive responses to facilitate the correlation and comparison of results with other studies.

In our study, when comparing the two groups, a statistically significant difference was found between the active tactile sensibility of the control group and the experimental group, on both the right and the left side, for thicknesses of 24 μm or higher, along with the perception of different thicknesses in the control group. This finding agrees with the study of Luraschi et al. [19] and the meta-analysis of Higaki et al. [25], which compared volunteers with complete natural dentition and volunteers rehabilitated with fixed mandibular prostheses.

Enkling et al. [8] affirmed that the perception of interocclusal sensibility allows the establishment of a parameter of the level of occlusal balance in which the patient does not feel a restoration with premature contact, and they also reported that this balance requires an accuracy of less than 100 μm, which is in agreement with the present study since the active tactile sensibility threshold of patients in the experimental group was 80 μm.

Higaki et al. [25] stated that the active tactile sensibility threshold of patients with implants is 1.2–2.3 times greater than in patients with natural dentition. Jacobs and van Steenberghe [18] concluded that the active tactile sensibility threshold is, on average, six times higher in patients with implants than in patients with natural teeth. In our study, considering both sides, the active tactile sensibility threshold was about 2.5 times higher for the prosthetic patients in comparison with the total dentulous ones.

A greater increase in perception in the control group on both sides as the carbon thickness increased was observed in comparison with the experimental group, in which this perception did not increase. This finding was based on bilateral analysis, and it agrees with recent studies stating that volunteers who have been rehabilitated with implant-supported prostheses present a higher active tactile sensibility threshold than volunteers with natural dentition [3,14,19]. However, to date, no other study has performed intraindividual assessments and reported differences regarding laterality for the same group [14,15,21]. Moreover, some studies have also shown that there is a difference in perception regarding the different types of materials used to fabricate the prosthesis. Henry et al. [13] stated that patients rehabilitated with ceramic-coated prostheses have lower thresholds, around 20 µm, than patients rehabilitated with prostheses with acrylic resin coating, whose thresholds are around 400 µm.

The oral perception of patients rehabilitated with implants requires time to improve active tactile sensibility. Abarca et al. [5] suggested that, whenever possible, complete rehabilitation should be done by keeping some teeth temporarily until sensibility improves. In this sense, studies assessing the active tactile sensibility of the same patient over time and a greater number of volunteers are also required.

The use of sheets of carbon might be considered a limitation of the present study since no other studies have used this material. Therefore, the authors could not make an adequate comparison with other data [3,14,15]. Another limitation inherent to this type of assessment is that it was not possible to match the muscular force of all volunteers during the bite [15,18] and to place the carbon paper at exactly the same position despite in the referenced methods being respected.

However, few studies of this kind used a control group of volunteers with complete natural dentition for comparison [3,19], and few studies in the literature [11,15,21] assessed the active tactile sensibility in patients with complete natural dentition. There is no precise report on the active tactile sensibility threshold in individuals with complete natural dentition and few studies on patients with complete implant-supported rehabilitations, which justifies the need for further studies on the subject.

## 5. Conclusions

Our findings confirm that there is a difference between the active tactile sensibility of patients who have been rehabilitated with fixed complete implants and those with complete natural dentition. We found an increase in perception of the control group on both sides as the carbon thickness increased. In addition, active tactile sensibility for the participants with mandibular implant-supported and maxillary mucosa-supported prostheses in comparison with dentate patients was significantly lower, detected above the thickness of 80 μm, without any differentiation of thicknesses.

## Figures and Tables

**Table 1:** Variables related to the study groups.

Variables	Control Group (*n* = 17)	Experimental Group (*n* = 18)
Age	52 ± 2.78	66 ± 8.67
Sex	-	-
Women	12 (70.6%)	12 (66.6%)
Men	5 (29.4%)	6 (33.4%)
Time of implant placement (years)	-	2 ± 1.7
Time of prosthesis placement (years)	-	4 ± 2.1

**Table 2:** Analysis of the differences in active tactile sensibility between groups.

Hemiarch	Thickness	Statistical Test (Mann–Whitney U Test)	*p*-Value
Right	12 µm	U = 121	0.288
	24 µm	U = 37.5	<0.0001
	40 µm	U = 13.5	<0.0001
	80 µm	U = 15.5	<0.0001
	200 µm	U = 36.5	<0.0001
Left	12 µm	U = 100	0.079
	24 µm	U = 49	0.0003
	40 µm	U = 22	<0.0001
	80 µm	U = 28	<0.0001
	200 µm	U = 44	<0.0001

## Data Availability

Not applicable.
